# Power and Area Efficient Cascaded Effectless GDI Approximate Adder for Accelerating Multimedia Applications Using Deep Learning Model

**DOI:** 10.1155/2022/3505439

**Published:** 2022-03-19

**Authors:** Manikandan Nagarajan, Rajappa Muthaiah, Yuvaraja Teekaraman, Ramya Kuppusamy, Arun Radhakrishnan

**Affiliations:** ^1^School of Computing, SASTRA Deemed University, Thanjavur 613 401, India; ^2^Department of Electronic and Electrical Engineering, The University of Sheffield, Sheffield S1 3JD, UK; ^3^Department of Electrical and Electronics Engineering, Sri Sairam College of Engineering, Bangalore 562 106, India; ^4^Faculty of Electrical & Computer Engineering, Jimma Institute of Technology, Jimma University, Jimma, Ethiopia

## Abstract

Approximate computing is an upsurging technique to accelerate the process through less computational effort while keeping admissible accuracy of error-tolerant applications such as multimedia and deep learning. Inheritance properties of the deep learning process aid the designer to abridge the circuitry and also to increase the computation speed at the cost of the accuracy of results. High computational complexity and low-power requirement of portable devices in the dark silicon era sought suitable alternate for Complementary Metal Oxide Semiconductor (CMOS) technology. Gate Diffusion Input (GDI) logic is one of the prompting alternatives to CMOS logic to reduce transistors and low-power design. In this work, a novel energy and area efficient 1-bit GDI-based full swing Energy and Area efficient Full Adder (EAFA) with minimum error distance is proposed. The proposed architecture was constructed to mitigate the cascaded effect problem in GDI-based circuits. It is proved by extending the proposed 1-bit GDI-based adder for different 16-bit Energy and Area Efficient High-Speed Error-Tolerant Adders (EAHSETA) segmented as accurate and inaccurate adder circuits. The proposed adder's design metrics in terms of delay, area, and power dissipation are verified through simulation using the Cadence tool. The proposed logic is deployed to accelerate the convolution process in the Low-Weight Digit Detector neural network for real-time handwritten digit classification application as a case study in the Intel Cyclone IV Field Programmable Gate Array (FPGA). The results confirm that our proposed EAHSETA occupies fewer logic elements and improves operation speed with the speed-up factor of 1.29 than other similar techniques while producing 95% of classification accuracy.

## 1. Introduction

The basic building blocks of any computational system are arithmetic circuits. In recent days, resource-constrained sensor-enabled less power-consuming devices such as mobile phones and embedded processors are used in various real-time machine and deep learning applications. Generally, our human visual and hearing system can tolerate some errors. Hence, for some of the signal processing applications, namely, speech, audio, image, and video processing, exact computation is not necessarily required. Moreover, integrated circuits for multimedia applications, particularly image and video processing which uses deep learning, take a large circuit area and high power due to the size of the data and computational complexity. Approximate computing is one of the most straightforward solutions to reduce circuit complexity and power consumption [1]. Therefore, this kind of inexact computing circuit is beneficial in low-power, resource-constrained devices, particularly for IoT and error-resilient applications such as multimedia and deep learning applications where exact computation is not that much significant. In addition to area and power, the delay is also reduced by using inexact computational circuits. Based on the idea, there are varieties of approximate adders (inexact adders) [2–9], and multiplier circuits [10–15] have been described in the literature for signal processing, image processing, and deep learning applications.

Carry-free arithmetic initiated with modified XoR-based approximate adder is presented in [2], and it is applied in image processing. Approximate mirror adders [3] of various configurations and truncated adders [4] are developed and used for image compression applications to verify their performance. Other similar types of inexact adders such as Significance Approximation Error-Tolerant Carry Select Adder (SAET-CSLA) based on Approximate Full Adder (AFA) [5], Modified Full Adder- (MFA-) based High-Speed Error-Tolerant Adder (HSETA) [6], and MUX-based High-Performance Error-Tolerant Adder (HPETA) [7] are designed, and better performance is demonstrated with image blending applications. Carry Truncate Adder (CTA) [8] is deployed in Convolution Neural Network (CNN) structure for accelerating the computation of the softmax layer. Generic Accuracy Configurable Adder (GeAr) is modified and implemented in the CNN-based Caffe network to accelerate the image to column conversion process [9].

Inexact adders are extended to form multipliers for the variant applications. Bioinspired Imprecise Computational (BIC) block adders and multipliers [10] are implemented for soft computing-based face recognition. Some of the other types of multipliers, namely, dynamic segmented multiplier [11], partial product perforated multiplier [12], compressor-based approximate multiplier [13], and truncation-based multiplier [14], are developed and evaluated for a variety of multimedia applications. The possible extent of loss of accuracy in the multiplier for neural network accelerator is analyzed by considering energy consumption in [15].

Most of the works mentioned above are extended for 16-bit operation. Fixed-point 16-bit arithmetic suits well for multimedia and deep learning applications. Optimized circuit structure for 16-bit arithmetic is of more interest. Generally, inexact computation uses a segmented approach. A 16-bit adder for the 16-bit word inexact computation can be divided into two halves such as 8-bit Least Significant Bit (LSB) portion and 8-bit Most Significant Bit (MSB) portion. As per the design convention, the LSB part where less weight information resides is designed using the approximate adder circuits, and the MSB part is designed using an accurate adder [5–7, 11, 16–19]. This segmented methodology minimizes the error and improves the circuit's performance in terms of area, power, and speed.

There is a limitation in attaining the area, power, and speed performances for deep learning systems in CMOS dark silicon era. Hence, a new technology that will trade off and achieve those performances is the need of the hour [20].

One such promising technology that we propose in this work to satisfy all the requirements of the real-time portable deep learning systems is Gate Diffusion Input (GDI) logic. The GDI logic is popular because it produces full swing output, which reduces the power consumption in digital circuits [21]. Hence, Gate Diffusion Input-based logic cells are trending and suitable alternate for the CMOS-based cells, especially for the low-power application. Also, the GDI logic-based design reduces the number of transistors used in the circuits [22] compared to the conventional CMOS logic design. Using GDI logic, many functions, including logic gates circuits, can be realized.

**Figure 1:**
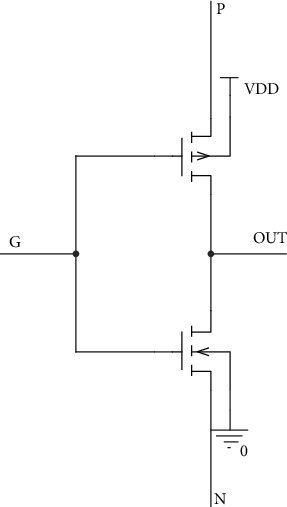
Basic GDI cell.

The primary GDI cell, which is shown in Figure 1, can perform six operations that include four fundamental and two special functions with the two MOSFET transistors, as listed in Table 1 [21].

GDI cells can be used to construct full adder circuits. However, while cascading multiple GDI cells to produce the sum and carry, the full swing problem is inevitable. To overcome this, a few more MOS transistors should be accompanied for having a full swing effect [22] at the expense of area and power.

One-bit adders are the building blocks of the 16-bit High-Speed Error-Tolerant Adders. Plenty of configurations of GDI-based full adders (1-bit adder) for low-power computing are found in literature [23–26]. GDI-based full adder for inexact computing is presented in [27]. The performance of an inexact full adder depends on its erroneous output and error distance [5–7]. Inexact circuit with minimal error and minimal error distance (ED) while using a smaller number of resources is challenging, and that meets the purpose of inexact computation. Our research contributes to a novel architecture that mitigates cascaded effects and addresses those challenges. Significant contributions in this work are as follows:

- It presents the architecture to address the cascading effect in GDI logic circuits.
- It proposes two full swing transistor-level EAFA architectures, namely, EAFA Design 1 with 10 transistors and EAFA Design 2 with 6 transistors.
- The proposed 1-bit EAFA is extended for 16-bit HSETA as Energy and Area efficient HSETA (EAHSETA), and two configurations of EAHSETA are proposed.
- The proposed EAHSETAI and EAHSETAII use GDI-based 4-bit CSLA and 4-bit Ripple Carry Adder (RCA) for its 8-bit MSB accurate part. For the inaccurate 8-bit part, the proposed full swing EAFA Design 1-based 8-bit adder and proposed full swing EAFA Design 2-based 8-bit adders are used, respectively.
- The proposed EAHSETA logic is implemented in the CNN-based Light Weight Digit Detector (LWDD) for accelerating handwritten digit classification application.
- FPGA implementation of the proposed logic-based accelerator is done on Intel Cyclone IV FPGA to verify the practicability of the proposed EAHSETA, and its performance as an accelerator is validated by comparing resource utilization, power, and speed of operation with other similar adders.

The rest of the manuscript is organized as follows. The methods and materials are given in Section 2. The design procedure of the proposed approximate adder circuits is detailed in Section 3. Experimental results and the performance comparison of the proposed design over other similar recent methods are presented in Section 4.  Section 5 describes the proposed adder logic as the CNN accelerator.  Section 6 concludes the paper.

## 2. Methods and Materials

There are numerous Approximate Full Adder circuits presented in the literature. Out of several inexact adders, more recent and minimum error distance-based adders are considered for discussion and comparison. An inexact adder, namely, Significance Approximate Error-Tolerant Adder (SAETA), is proposed to minimize the number of logic gates [5]. The proposed approximate adder circuit produces two errors in sum output and no error in carry output. The proposed SAETA is used to design a 16-bit error-tolerant circuit that uses common CSLA for accurate part and SAETA in inaccurate part. The performance of the adder in terms of area, power, and delay is compared with that of common and other inexact adders. In addition, the designed adder was tested using image processing applications.

In [6], the authors proposed a 1-bit Modified Full Adder (MFA) with less error distance. The one-bit MFA is extended to design a 16-bit HSETA that uses common CSLA for higher-order 8 bits (i.e., for 8 MSB bits) and their MFA for lower-order 8 bits (i.e., for 8 LSB bits). The presented design is justified and compared experimentally with the standard and recent related works based on area, power, and delay performance. Though it confers better results compared to others, its normalized error distance is more.

Recently, to overcome the voltage swing problem of CMOS logic in [27], the author presented a Modified Full Adder [6] with GDI logic using 14 and 12 transistors designs. It claims better area and power performances compared to a common adder at the cost of increased error distance. The circuit is simulated in the Cadence Design suite, and logic is realized in FPGA.

The hybrid method has been deployed by combining multithreshold voltage transistor logic with GDI logic to overcome the full swing issue [28]. The presented full adder uses 14 transistors to produce accurate results, and the proposed full adder is extended to the 32-bit adder. Even though the adder's accuracy is much better, the area occupied by design is still higher than that of other recent works.

**Figure 2:**
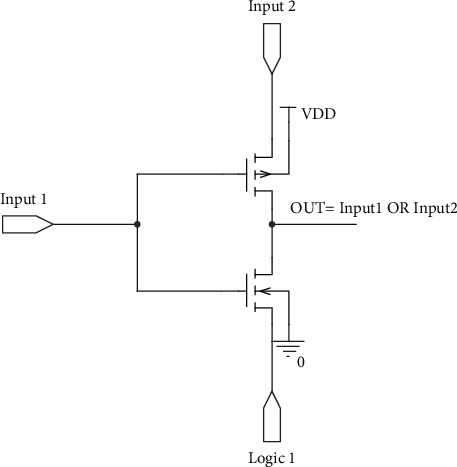
GDI-based OR gate.

**Figure 3:**
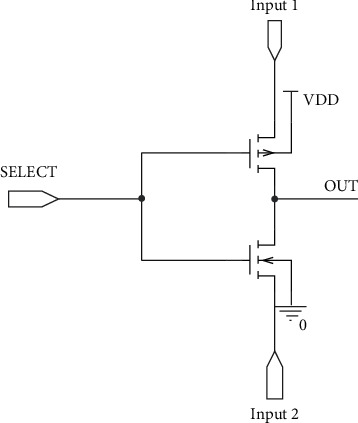
GDI-based MUX.

**Figure 4:**
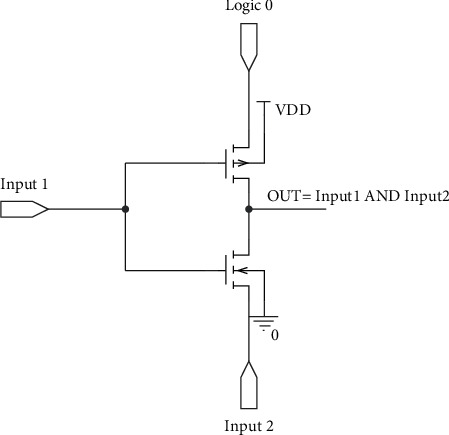
GDI-based AND gate.

In order to compare our proposed full swing inexact adder results with accurate adder results, the low-power full adder circuit presented in [22] with GDI logic is taken along with AFA and MFA. The truth table of the same is listed in Table 2, and the injected errors are highlighted. In this work, initially, primary gate circuits such as AND, Multiplexer, and OR using two different functions based on GDI logic are designed and are shown in Figures 2–4, respectively. Later two different full swing full adder circuits have been designed based on the primary blocks with a lesser number of transistors.

## 3. Proposed GDI-Based Adders

In this section, two proposed error-tolerant EAFA designs featuring GDI with full swing logic are discussed with the aim of reducing the circuit area and power and attaining speed at multibit addition operation. The proposed EAFA design minimizes the error distance with reduced circuit area (less transistor count), power, and delay compared to the similar work presented in [5, 6] with two errors.

In Table 3, Boolean terms of common and 1-bit Error-Tolerant Adders with two errors are listed. From the expressions, it is evident that a sum expression of the common adder, AFA, and MFA uses cascaded logic gates for the adder logic realization. The cascaded logic gates reduce the voltage swing level in GDI logic implementation. This eventually needs full swing implementation for the proper sum output, and interns increase the area of implementation through transistor count.

Except MFA, the remaining existing adders carry expression also uses the cascaded logic gates and leads to the aforesaid problem. Our proposed full swing EAFA adder carefully avoids cascaded logic, and it uses the AND, OR, and Multiplexer (MUX) functionalities of the GDI logic cell to realize the sum and directly takes input A as the carry output. This design implements the same kind of 1-bit adder of two errors with minimal transistor delay and power compared to others. Here, Multiplexer plays a vital role in all the minimization.

**Figure 5:**
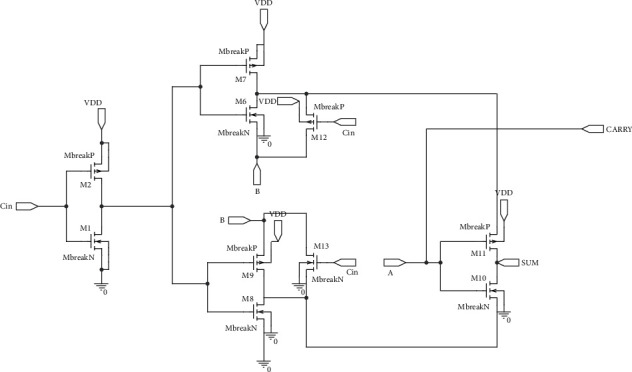
GDI-based full swing EAFA Design 1 with 10 transistors.

**Figure 6:**
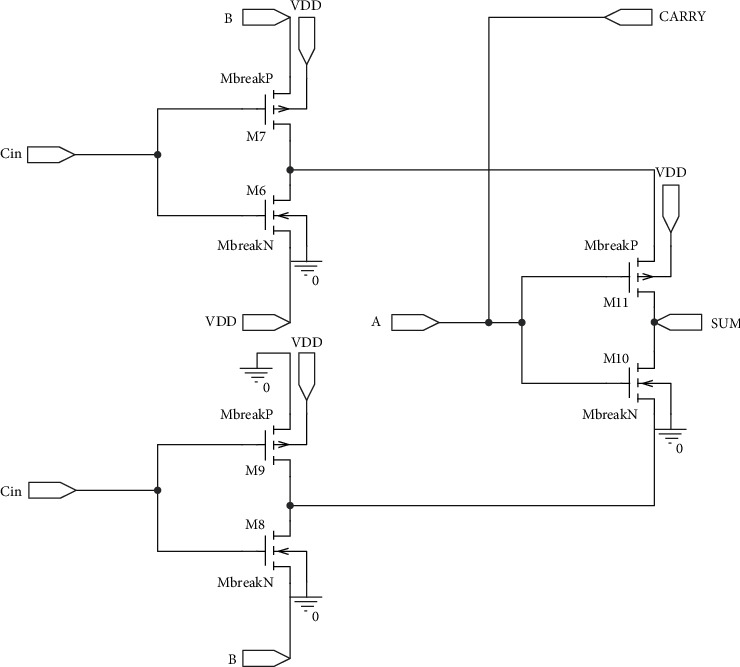
GDI-based full swing EAFA Design 2 with 6 transistors.

EAFA Design 1 is implemented with 10 transistors using full swing AND and OR gates, as shown in Figure 5. EAFA Design 2 is deployed with 6 transistors, which uses standard AND and OR gates along with Multiplexer to produce the full swing output. This ability of full swing with a smaller number of transistors is achieved by the noncascaded structure of the proposed circuit, which is presented in Figure 6.

### 3.1. Segmented Approximate Adders

Approximate adders are the core and essential part of any approximate circuits used for processing the signals or data. Segmented approximate addition is the most widely used method for its accuracy and error trade-off [5–7, 11, 16–19]. In segmented approximate adders, half of the binary data from the MSB and half of the binary data from the LSB are segmented separately. Upper MSB segment data is added by the accurate adder to preserve the quality of the results, and lower LSB segment data is added by an inaccurate adder, which aids in the speed and energy efficiency of computation. In the existing 16-bit GDI High-Speed Error-Tolerant Adder (GDI-HSETAI and II) [27], authors suggested the 8-bit CSLA for the 8-bit accurate part and inaccurate adders for lower 8 bits.

### 3.2. Proposed Segmented Approximate Adders

**Figure 7:**
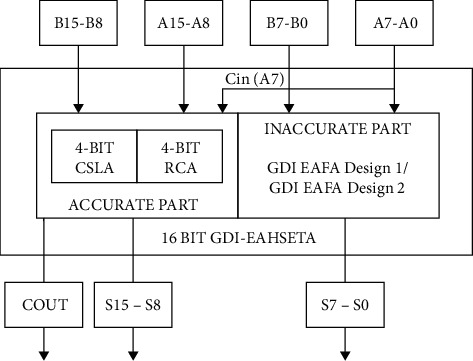
Block diagram of the proposed 16-bit GDI-EAHSETA.

In our work, we proposed two 16-bit GDI Energy and Area Efficient High-Speed Error-Tolerant Adders (GDI-EAHSETA) as described in Figure 7 based on our 1-bit adder EAFA Design 1 and EAFA Design 2. In the proposed 16-bit adder, we modified the accurate 8-bit adder, which uses a combination of 4-bit CSLA and 4-bit RCA [29–32] for the upper MSB 8-bit segment. Lower 8-bit LSB segment uses our proposed EAFA designs.

**Figure 8:**
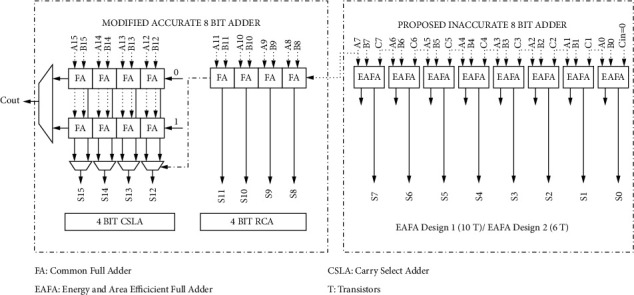
Detailed block diagram of the proposed 16-bit GDI-EAHSETA.

From the detailed proposed block diagram presented in Figure 8, it is evident that our proposed architecture has only 12 numbers of common full adders and 5 multiplexers in the accurate 8-bit adder, and the optimized design portion is highlighted in the same diagram. Our modified accurate 8-bit adder in the 16-bit GDI-EAHSETA uses 25% less number of FAs and 50% less number of multiplexers compared to the existing 16-bit GDI-HSETA. EAFA Designs 1 and 2 used in the inaccurate 8-bit adder consist of 10 and 6 transistors, respectively. Those proposed EAFA designs occupy 16.67% and 50% less area, respectively, in comparison with the efficient existing MFA Design 2 inaccurate 8-bit adders.

## 4. Experimental Results and Discussion

All the circuits presented in this work have been simulated with the Cadence EDA tools with 90 nm PTM.

### 4.1. Proposed 1-Bit Full Swing EAFA

Both the proposed full adders exhibit full swing performance. In the EAFA Design 1, full swing AND and OR GDI logic gate is used to maintain the voltage level, and it is given through the GDI MUX for selecting the proper sum value of the given input.

**Figure 9:**
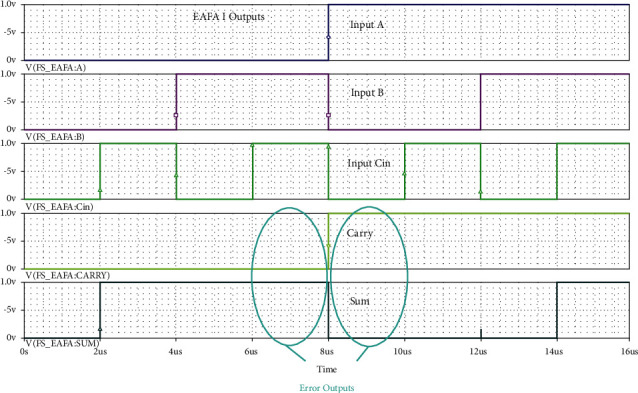
Simulation results of the proposed 10-transistor 1-bit full swing EAFA Design 1 for various input combinations.

**Figure 10:**
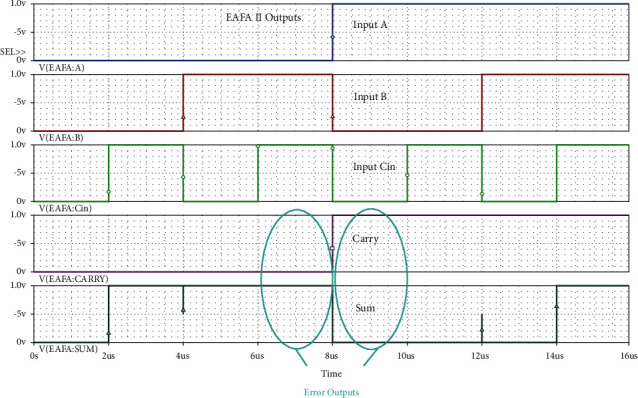
Simulation results of proposed 6-transistor 1-bit full swing EAFA Design 2 for various input combinations.

Full swing structure of AND and OR there itself manages the signal levels not to go down below and above the specific voltage level to represent logic 0 and 1. MUX simply passes the logic value through it to the sum output. Its simulated results for all the input combinations are shown in Figure 9. From simulation results of Figures 9 and 10, it is evident that there are no voltage level moderations in the sum and carry outputs of both the adders. Our proposed 6-transistor EAFA Design 2 itself generates full swing output without any extra transistors for modifying the voltage levels at par with the common adder, AFA, MFA, and EAFA Design 1.

### 4.2. Power Consumption and Delay

The average power consumption is computed through the measurement tool in the SPICE simulation. The maximum of the average power is calculated and taken as a consumed power of the adder circuits [27].

The delay of all the adders is measured by calculating the time difference between the time taken by the input voltage swing to rise or fall from its 50% of the maximum value. The maximum delay got from various input and output combinations is taken as the worst-case delay [24]. The power and delay results of the various simulated adders are presented in Table 4.

From the simulated results of all the 1-bit full adders, it is prompted that our proposed EAFA Design 1 has consumed 47.62% less power compared to best among common adders (Design 1) and 46.15% less power compared to best among AFA adders (Design 2) and 44.42% less power compared to best among MFA adder (Design 2).

**Figure 11:**
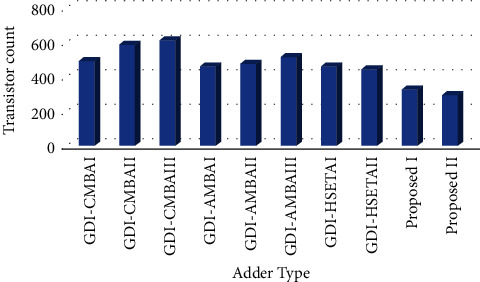
Comparison of the area of various 16-bit adders with the proposed GDI-EAHSETA adders in terms of transistor count.

In the same way, our proposed EAFA Design 2 outperforms other adders with 99.99% less power consumption. This power performance has been achieved in our proposed structure by means of handling switching activity involved in producing the sum and carry terms. For any combination of input, in a given time, only three transistors are in ON state for producing a sum output, and for generating carry, transistors need not spend energy since it is directly taken from one of the inputs. The speed performance of the proposed EAFA adders is good with a minimal delay compared to other simulated adders. Our EAFA Design 1 has reduced the delay of 38.79%, 41.16%, and 30.66%, compared to corresponding best performing adders in their groups, namely, common adder Design 1, AFA Design 2, and MFA Design 2, respectively. The worst-case delay of 459.447 ps is measured between the sum and the input A value. The proposed EAFA Design 2 has 86.7%, 87.21%, and 84.93% less delay compared to the common adder Design 1, AFA Design 2, and MFA Design 2, respectively, and the same is illustrated in Figure 11.

The worst-case delay of the adder has arrived between the input A and the sum output, and its value is 99.866 ps. The reason behind the minimal delay is that both the proposed designs need not spend time in carry calculation, and hence carry does not have an effect over the delay. The sum output is the dominant player in the delay. In our proposed Design 2, to generate the sum for any combination of input, the signal has to travel through two numbers of transistors only. This makes sense for the speed of operation of our proposed circuit architecture.

### 4.3. Performance Evaluation of 16-Bit Adders

In order to prove the cascaded effectless operation of the proposed adder, 1-bit EAFA Design 1 and Design 2 are extended to form 16-bit adders and are compared with various configurations of 16-bit adders of different types as listed in Table 5. The total area occupied by each type in terms of transistor count and power consumed by the corresponding adders is also listed. GDI Common Multibit Adders (GDI-CMBA) I to III use the accurate adders, and GDI Approximate Multibit Adders (GDI-AMBA) I to III are formed by combining accurate adder for MSB 8 bits and inaccurate AFA for LSB 8 bits. GDI-HSETAI and II are created by an 8-bit accurate adder and 8-bit inaccurate MFA adder. In the first three types, GDI-CMBA occupies less area and consumes less power. From type 4 to type 8 adders, GDI-AMBAII performs well among AFA-based designs, and GDI-HSETAII performs well among MFA-based designs.

Among the listed adders in Table 5, our proposed 16-bit GDI-EAHSETAI occupies 12 × 18 = 216 transistors for 12 common full adder Designs 1, 5 × 6 = 30 transistors for 5 multiplexers, and 8 × 10 = 80 transistors for inaccurate 8-bit adder based on EAFA Design 1. All put together, it occupies 326 transistors (216 + 30 + 80) only. That is less than 33.74%, 31.51%, and 26.57% area of relatively well-performing adders, namely, GDI-CMBAI, GDI-AMBAII, and GDI-HSETAII adders, respectively. Similarly, our proposed 16-bit GDI-EAHSETAII occupies a total of 294 transistors which are summed up from 12 × 18 = 216 transistors for common full adders, 5 × 6 = 30 transistors for multiplexers, and 8 × 6 = 48 transistors for inaccurate EAFA Design 2-based inaccurate adder. The area occupied by the GDI-EAHSETAII adder is 40.24%, 38.23%, 33.78%, and 9.8% less than the area needed to implement the GDI-CMBAI, GDI-AMBAII, GDI-HSETAII, and GDI-EAHSETAI, respectively. A comparison of the area occupied by the proposed high-speed adder with other adders is illustrated in Figure 11.

**Figure 12:**
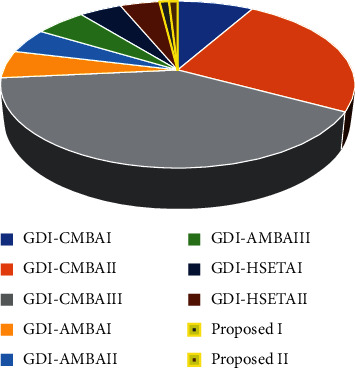
Comparison of power consumption of various 16-bit adders with the proposed GDI-EAHSETA adders.

The average power has been measured for all the 16-bit adders using Cadence SPICE. The substantial reduction in the number of transistors and the circuit structure aids less power consumption of our proposed 16-bit GDI-EAHSETAI and II. In comparison with other 16-bit adders proposed, GDI-EAHSETAI consumes 87.5%, 78.92%, and 75.78% less power, GDI-EAHSETAII consumes 88.31%, 80.27%, and 77.34% less power than GDI-CMBAI, GDI-AMBAII, and GDI-HSETAII, respectively, and this is illustrated in Figure 12. Our proposed GDI-EAHSETAII outperforms all the types of listed adders by means of area and power while producing distortion-free outputs.

### 4.4. Performance Evaluation of the Adders in FPGA

For the practicability and to evaluate the proposed logic performance, we have implemented the best inaccurate-based 16-bit adder logic among all ten types, which are synthesized using Quartus Prime 18.0 tool for the Intel Cyclone IV FPGA platform, and parameters are listed in Table 6. In this hardware platform also, our proposed adder outperforms other adders by occupying fewer LUTs, consuming less power, and with improved speed of operation.

### 4.5. Error Characteristic of Approximate Adders

Approximate adders are characterized by their nonconformity of calculated results from the precise results. Various error parameters such as error distance (ED) and mean error distance (MED) are used to properly characterize the approximate adders for practical applications. Error distance is the absolute difference of the added results with definite results. Mean error distance is calculated as the average of all the error distances of an inaccurate adder circuit, and it is given in equation (1). In order to make an additional comparison with similar adders, one more parameter is derived from MED called normalized mean error distance (NMED). NMED is calculated as in equation (2) as the ratio of MED to the maximum error value (*D*) of that specific adder.

(1) $$\begin{array}{c} \text{MED}=\frac{1}{2^{2n}}\sum_{i=1}^{2^{2n}}ED_{i}, \end{array}$$

(2) $$\begin{array}{c} \text{NMED}=\frac{\text{MED}}{D}. \end{array}$$

Error characteristics are useful to validate the approximate adders for their suitability for application deployment. Idea MED of accurate adder is “0.” So, an approximate adder produces the MED value nearing the ideal value, which is measured as good for use in computation applications. Similarly, NMED is the derived parameter from MED to sense the overall error distribution with respect to the maximum possible error in the designed approximate adder.

Based on equations (1) and (2), error metrics have been calculated and tabulated in Table 7. From this, our proposed added adder exhibits better error characteristics compared to other existing adders, and it is very well suited for real-time error-resilient applications such as deep learning-based image processing.

## 5. Convolutional Neural Network Accelerator for the Handwritten Digit Classification Inference: A Case Study

Convolutional neural networks are drawing major attention in deep learning-based applications, especially classification and detection [33, 34]. Even CNN is also used to predict high-frequency details lost in low-resolution images to create superresolution images [35].

CNNs are inheriting error tolerance through their learning and updating process of weights by random initialization. In this work, we took the privilege of the error-resilient property of CNN and focused on developing the most common block, which is used in the convolution computation. The fundamental block frequently involved in all the computation processes of CNN is the adder. Adder contributes to the whole system's performance and influences the total energy consumption. Thus, introducing the inexactness in the addition process curtails the power, area, and delay while improving the whole system's performance [8, 9].

Since CNN is the most commonly used method in any deep learning process, we attempted to accelerate the computationally intensive convolutional blocks in it. In this work, an accelerator to accelerate the CNN for handwritten digit classification application is developed through dedicated fixed-point approximate arithmetic blocks for the convolution operation. A similar kind of accelerator work can be found in the literature, but for accelerating softmax regression [8] and image to column operation [9] alone.

### 5.1. CNN System Architecture for Digit Classification

**Figure 13:**
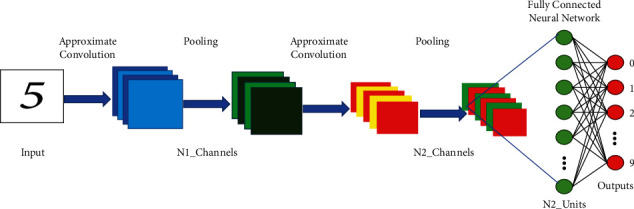
Proposed approximate convolution in the typical CNN architecture.

A CNN is a multilayer filter specially designed to visualize data information through preprocessing operations. In CNN, the input parameter size has been decreased layer by layer at the same time the size of the filter increases. The general system architecture of CNN for digital identification is shown in Figure 13. As the depth of the network increases, that is, the number of layers in between the input and the output increases, the accuracy of the digit classification also increases [36].

### 5.2. VGG Net

In this work, we have concentrated only on VGG-based Low-Weight Digit Detector (LWDD) CNN system architecture, which is deployed in real-time handwritten digit recognition applications to detect digits from 0 to 9 [37].

In LWDD, authors optimized various layers and made the weights as a 16-bit fixed-point to minimize the total size and count of the weight parameters. These modifications have greatly reduced the storage requirements of the parameters, which made them easily fit in the FPGA. Different layers structure and the corresponding sizes of the images and parameters of the same in the LWDD are given in Table 8.

This LWDD architecture gets 28 × 28 size handwritten digit images as input and classifies it as a number between 0 and 9. Handwritten digit images from the open MNIST dataset are used for training the network, and the trained parameters are used for inferencing digit classification.

### 5.3. Performance of Evaluation of the Proposed Adder in CNN Accelerator

In order to accelerate the real-time handwritten digit recognition process, we replaced conventional adders used in the convolution layers of the LWDD architecture with our proposed GDI-EAHSETA approximate adder logic and evaluated.

**Figure 14:**
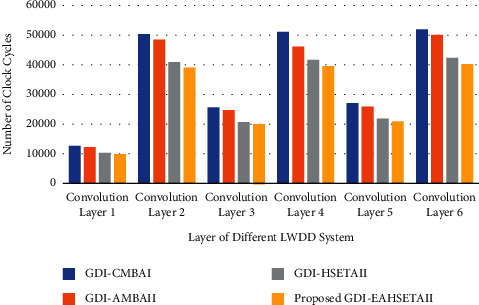
Clock cycles taken by different levels of convolution layer for different systems.

For a fair comparison of the acceleration process, the existing competing adders GDI-AMBA and GDI-HSETA are also implemented in the accelerator. The hardware complexity for processing convolution layer is much simpler in our proposed architecture, the comparison of time taken for calculation at different layers in terms of clock cycles is listed in Table 9, and its graphical representation is presented in Figure 14.

From the comparison, it is evident that our proposed GDI-EAHSETAII greatly reduces the number of clock cycles needed for the convolution compared to conventional accurate adder GDI-CMBAI and speeds up the process by the factor of 1.29. In comparison with related approximate adders, the proposed adder exhibits relatively better performance by taking a smaller number of clock cycles. Comparison of clock cycles taken by the different levels of convolution layers for the various LWDD system deployed different approximate adders.

FPGA implementation of LWDD accelerator system based on various adders is done in Intel Cyclone IV EP4CE22F17C6N FPGA, and the resources utilized for the deployment of the accelerator are listed in Table 10 along with the digit classification accuracy. GDI-AMBAII utilizes more resources compared to other approximate adders with the speed-up factor of 1.04, but still, it produces moderate accuracy of 88%. GDI-HSETAII consumes moderate resources while showing very little accuracy of 80% among others, even though it speeds up the computation process by the factor of 1.23. The proposed GDI-HSETAII utilizes a smaller resource while producing 95% accuracy; it speeds up the computation process by 1.29 factors. Since the proposed adder is outperforming all other adders in terms of resource utilization, speed, and accuracy, it is best suited for the CNN inference accelerator.

## 6. Conclusion

In this work, we designed and evaluated the transistor-level 16-bit Energy and Area Efficient High-Speed Error-Tolerant Adders based on the proposed GDI-based full swing energy and area efficient error-tolerant full adders for handling the cascading effect. In comparison with the similar kind of inexact full adders, the proposed EAFA Design 1 and EAFA Design 2 have a lesser area, consuming less power and better speed of operation while giving better reliability with the minimum error distance. The efficiency of the proposed EAHSETA is compared with the various multibit adders based on common CSLA designs, AFA, MFA, and HSETA. The improvements in the speed, area, power of EAHSETAI and EAHSETAII are achieved through the cascaded effectless architecture of the EAFA designs. The proposed EAHSETAII has a relatively lesser area and consumes 88.31%, 80.27%, and 77.34% lesser power than in-group best performing GDI-CMBAI, GDI-AMBAII, and GDI-HSETAII, respectively. These features of EAHSETAII satisfy the area and power requirements of resource-constrained high-speed, low-power deep learning applications. The proposed EAHSETAII, AMBAII, and HSETAII logics are deployed to accelerate the convolution computation of the LWDD network, which is used in real-time handwritten digit recognition. All the systems are implemented in the Intel Cyclone IV FPGA, and performance has been evaluated. The proposed EAHSETAII outperforms other similar logics by producing 95% classification accuracy with the speed-up factor of 1.29 while consuming fewer resources. Experimental results also confer that AMBAII is able to produce moderate accuracy with less speed and HSETAII logic exhibits poor accuracy with the moderate speed-up factor. In the future, the proposed logic can be extended to an inexact multiplier design for the multiply and accumulate unit for further acceleration.

## Figures and Tables

**Table 1:** Truth table of basic GDI cell.

*N*	*P*	*G*	Out	Function
“0”	*B*	*A*	*A*'*B*	*F*1
*B*	“1”	*A*	*A*' + *B*	*F*2
“1”	*B*	*A*	*A* + *B*	OR
*B*	“0”	*A*	*AB*	AND
*C*	*B*	*A*	*A*'*B* + *AC*	MUX
“0”	“1”	*A*	*A*'	NOT

**Table 2:** Truth table of proposed error-tolerant design with minimal error distance.

Inputs			Accurate output [18]		Adder [5]			Adder [6]			Proposed adder		
					AFA			MFA			EAFA		
*A*	*B*	*C*_in_	*C*_out_	*S*	*C*_out_	*S*	ED	*C*_out_	*S*	ED	*C*_out_	*S*	ED
0	0	0	0	0	0	1	+1	0	0	0	0	0	0
0	0	1	0	1	0	1	0	0	1	0	0	1	0
0	1	0	0	1	0	1	0	0	1	0	0	1	0
0	1	1	1	0	1	0	0	0	0	−2	0	1	−1
1	0	0	0	1	0	1	0	1	1	+2	1	0	+1
1	0	1	1	0	1	0	0	1	0	0	1	0	0
1	1	0	1	0	1	0	0	1	0	0	1	0	0
1	1	1	1	1	1	0	-1	1	1	0	1	1	0

**Table 3:** List of Boolean expressions to implement the accurate and error-tolerant full adder designs.

Type		Sum	Carry
Common adder [18]	Design 1	$\text{Sum}=\bar{C_{\text{in}}\;}\left(A\text{XOR}B\right)+C_{\text{in}}\left(A\text{XOR}B\right)$	$C_{\text{Out}}=\left(A\text{XOR}\;B\right)C_{\text{in}}+\bar{\left(A\text{XOR}B\right)}$
	Design 2	Sum=*A*XOR*B*XOR*C*_in_	$C_{\text{Out}}=\bar{C_{\text{in}}}\left(A\text{AND}B\right)+C_{\text{in}}\left(A\text{OR}B\right)$
	Design 3	Sum=*A*XOR*B*XOR*C*_in_	*C*_Out_=*A*AND*B*+ (*A*XOR*B*)*C*_in_
AFA [5]	Design 1	Sum=NOT(*C*_Out_)	$C_{\text{Out}}=\left(A\text{XOR}B\right)C_{\text{in}}+\bar{\left(A\text{XOR}B\right)}$
	Design 2	Sum=NOT(*C*_Out_)	$C_{\text{Out}}=\bar{C_{\text{in}}}\left(A\text{AND}B\right)+C_{\text{in}}\left(A\text{OR}B\right)$
	Design 3	Sum=NOT(*C*_Out_)	*C*_Out_=*A*AND*B*+ (*A*XOR*B*)*C*_in_
MFA [6]	Design 1	$\text{Sum}=\bar{C_{\text{in}}}\left(A\text{XOR}B\right)+C_{\text{in}}\left(A\text{XOR}B\right)$	*C*_Out_=*A*
	Design 2	Sum=*A*XOR*B*XOR*C*_in_	*C*_Out_=*A*
EAFA (proposed adder)	Design 1	$\text{Sum}=\bar{\text{A}}\left(B\text{OR}C_{\text{in}}\;\right)+A\left(B\text{AND}C_{\text{in}}\right)$	*C*_Out_=*A*
	Design 2	$\text{Sum}=\bar{\text{A}}\left(B\text{OR}C_{\text{in}}\;\right)+A\left(B\text{AND}C_{\text{in}}\right)$	*C*_Out_=*A*

**Table 4:** Simulation results of various types of 1-bit FA cells.

Sl. no.	Types		Power dissipation (W)	Delay (ps)	No. of transistors
1	Common adder	Design 1	649.966*µ*	750.649	18
2		Design 2	811.491*µ*	983.811	22
3		Design 3	988.816*µ*	992.583	23
4	AFA	Design 1	812.450*µ*	850.435	14
5		Design 2	632.276*µ*	780.870	16
6		Design 3	1057.88*µ*	810.526	21
7	MFA	Design 1	649.966*µ*	724.348	14
8		Design 2	612.572*µ*	662.609	12
9	**Proposed EAFA**	**Design 1**	**340.476*µ***	**459.447**	**10**
10	**Proposed EAFA**	**Design 2**	**175.336p**	**99.866**	**6**

**Table 5:** Comparison of various 16-bit adder configurations in terms of transistor count and power dissipation.

Sl. no.	Type	16-bit adder configurations		Count of adder cells and transistors			Multiplexers		Total (1) + (2)	Power dissipation (mW)
		Accurate (8-bit MSB part)	Inaccurate (8-bit LSB part)	Accurate (8-bit MSB part)	Inaccurate (8-bit LSB part)	Total no. of transistors (1)	No. of 2 : 1 MUX	Total no. of transistors (2)		
1	GDI-CMBAI	Common Design 1 (CSLA)	Common Design 1	16	8	432	10	60	492	24.8
2	GDI-CMBAII	Common Design 1 (CSLA)	Common Design 2	16	8	528	10	60	588	74.4
3	GDI-CMBAIII	Common Design 1 (CSLA)	Common Design 3	16	8	552	10	60	612	121.4
4	GDI-AMBAI	Common Design 1 (CSLA)	AFA Design 1	16	8	400	10	60	460	15.95
5	GDI-AMBAII	Common Design 1 (CSLA)	AFA Design 2	16	8	416	10	60	476	14.7
6	GDI-AMBAIII	Common Design 1 (CSLA)	AFA Design 3	16	8	456	10	60	516	16.54
7	GDI-HSETAI	Common Design 1 (CSLA)	MFA Design 1	16	8	400	10	60	460	14.0
8	GDI-HSETAII	Common Design 1 (CSLA)	MFA Design 2	16	8	384	10	60	444	12.8
9	**(Proposed) GDI-EAHSETAI**	**Common Design 1 (4-bit CSLA + 4-bit RCA)**	**EAFA Design 1**	**12**	**8**	**296**	**5**	**30**	**326**	**3.1**
10	**(Proposed) GDI-EAHSETAII**	**Common Design 1 (4-bit CSLA + 4-bit RCA)**	**EAFA Design 2**	**12**	**8**	**264**	**5**	**30**	**294**	**2.9**

**Table 6:** Synthesized results of the existing and proposed approximate adders.

16-Bit adder design	Logic cells	Power (mW)	Delay (ns)
GDI-CMBAI	32	113.22	19.351
GDI-AMBAII	32	113.22	18.904
GDI-HSETAII	24	111.71	15.758
Proposed GDI-EAHSETAII	20	108.54	14.755

**Table 7:** Error characteristics of existing and proposed approximate adders.

16-bit adder design	MED	NMED
GDI-AMBAII	0.0064	4.88 × 10^−8^
GDI-HSETAII	0.0095	7.25 × 10^−8^
Proposed GDI-EAHSETAII	0.0048	3.66 × 10^−8^

**Table 8:** Low-Weight Digit Detector network architecture.

Layer (type)	Output shape	Parameter
Input_1 (inputLayer)	(28, 28, 1)	0
Conv1 (conv2D)	(28, 28, 4)	36
Activation_1 (activation)	(28, 28, 4)	0
Conv2 (conv2D)	(28, 28, 4)	144
Activation_2 (activation)	(28, 28, 4)	0
Pool1 (maxpooling2D)	(14, 14, 4)	0
Conv3 (conv2D)	(14, 14, 8)	288
Activation_3 (activation)	(14, 14, 8)	0
Conv4 (conv2D)	(14, 14, 8)	576
Activation_4 (activation)	(14, 14, 8)	0
Pool2 (maxpooling2D)	(7, 7, 8)	0
Conv5 (conv2D)	(7, 7, 16)	1152
Activation_5 (activation)	(7, 7, 16)	0
Conv6 (conv2D)	(7, 7, 16)	2304
Activation_6 (activation)	(7, 7, 16)	0
Global_max_pooling2d_1 (global)	(16)	0
Dense_1 (dense)	(11)	176
Activation_7 (activation)	(11)	0

**Table 9:** Comparison of clock cycles taken for computation by the convolution layers in the LWDD network.

Stage of processing	Number of clock cycles				Speed-up factor		
	LWDD system based on different adders						
	GDI-CMBAI	GDI-AMBAII	GDI-HSETAII	Proposed GDI-EAHSETAII	GDI-AMBAII	GDI-HSETAII	Proposed GDI-EAHSETAII
Convolution layer 1	12605	12120	10245	9771	1.04	1.23	1.29
Convolution layer 2	50416	48470	40990	39112	1.04	1.23	1.29
Convolution layer 3	25569	24587	20789	19977	1.04	1.23	1.29
Convolution layer 4	51136	46171	41560	39609	1.04	1.23	1.29
Convolution layer 5	27009	25976	21956	20885	1.04	1.23	1.29
Convolution layer 6	52016	50015	42290	40320	1.04	1.23	1.29

**Table 10:** Resource utilization by the LWDD system and accuracy of classification.

LWDD system	Logic elements	Memory	Embedded multiplier 9-bit elements	Critical path delay (ns)	Classification accuracy (%)
GDI-CMBAI	3750	232111	25	21.840	96
GDI-AMBAII	3722	232111	25	21.720	88
GDI-HSETAII	3410	200700	23	20.570	80
Proposed GDI-EAHSETAII	3275	200221	23	20.332	95

## Data Availability

The data used to support the findings of this study are available from the corresponding author upon request.
